# London Post-Graduate Institutions

**Published:** 1911-09-02

**Authors:** 


### LONDON POST-GRADUATE INSTITUTIONS.

### WEST LONDON POST-GRADUATE COLLEGE.

The West London Hospital, Hammersmith, was the first of the general hospitals in London to reserve its practice strictly for qualified men, and to establish a post-graduate College as an integral part of its constitution. The hospital itself contains 160 beds, and the advantages of the direct connection of the College with the wards and out-patient work are obvious. It is recognised by the Royal College of Surgeons as an institution where candidates (who need not be members) for the Fellowship can spend the necessary year of study after obtaining a registrable qualification, while the College and hospital are also recognised by the naval and military authorities as regards study leave. The hospital is authorised by the University of London to give certificates of post-graduate study for the M.D. and M.S degrees.

Members of the College may also accompany the resident staff on their visit to the wards, and so have opportunities of refreshing and extending their knowledge of clinical methods. Operations are performed daily, at 2.30 p.m. Post-graduates are allowed to stand near the table and can see the operations perfectly. The surgeons often avail themselves of the assistance of post-graduates at operations.

The out-patient departments necessarily resemble in their main features those of other general hospitals, and the staff are always alert to discover, and, when found, to demonstrate, any cases of exceptional interest, or, what is of equal importance, any points of special interest found in cases of the more common diseases. During the sessions lectures are given daily, except Saturdays, at 5 p.m., on practical medicine and surgery in all their branches. There are also short courses on tropical diseases, and on mental diseases by men who have made their mark in those subjects.

Small classes are formed, when required, in connection with diseases of special regions, such as eye, throat, skin, etc.; for the clinical examination of blood and urine; in vaccine therapy, bacteriology and medical microscopy generally; in applied anatomy, administration of anæsthetics, intestinal surgery, cystoscopy, $x$-ray work, etc. For these classes, each of which is strictly limited to a small number, additional fees are required, as they are practically individual and tutorial. The ordinary fee for membership covers attendance at all general lectures and at all the routine practice of the hospital, whether in the general or special wards or departments. Autopsies can, of course, be attended. Arrangements can be made through the College authorities for special coaching should it be desired.

Members may join for any period from one week upwards, the fees ranging from one guinea to £30.

### THE LONDON POLYCLINIC.

The Medical Graduates' College and Polyclinic, founded in 1899, mainly through the efforts of Mr. (now Sir) Jonathan Hutchinson, has now a well-recognised position among the medical educational organisations of the metropolis. Its object is to provide opportunities for clinical and practical study for those who have already gained a position on the medical register. From the outset two principles have been recognised as essential to a large and broad scheme of post-graduation study. First, that such a scheme should be entirely separate and distinct from the ordinary educational course of the medical student. Secondly, that the teaching should not be confined to the staff of any individual hospital, but should include capable and distinguished representatives of all schools. Hence, at the Polyclinic, the needs of the practitioner receive full consideration, and the teaching in a very special manner represents the work of many of the best-known physicians and surgeons in the metropolis.

The afternoon cliniques are conducted daily at four o'clock, and offer valuable opportunities. Medicine, surgery, and each of the more special branches of practice, has its appropriate day. Selected cases are demonstrated and made the subject of clinical comment, and opportunities for personal examination by those attending the clinique are offered. In this way, and in a comparatively short period of time, the practitioner is able to overtake a large quantity of interesting and instructive clinical material under the direction of teachers specially qualified in each of the various departments. Indeed, it is difficult to imagine any scheme more suitable for those who are anxious to increase their clinical experience both in general and special work. On four days of each week there is also a lecture, illustrated by lantern slides, pathological specimens, or clinical cases, by a teacher of repute from one of the provincial or metropolitan schools. In these lectures the practical note is maintained throughout.

With a view to render these opportunities accessible to all, the annual subscription, which includes admission to all cliniques and lectures and also the use of the library and museum has been fixed at one guinea. In addition, the *Polyclinic Journal* is issued monthly, and is sent free to all subscribers. In the College laboratory specimens are examined and reported on for small fees, and here practical personal instruction in all the branches of clinical pathology can be obtained. Another part of the Polyclinic scheme, and one of great importance, is the provision of "practical classes" in the more recent methods of clinical investigation. Included in this division, instruction is provided in the use of the ophthalmoscope, laryngoscope, otoscope, cystoscope, sigmoidoscope, and other specialised clinical instruments; in the clinical examination of the blood, sputa, urine, etc.; in the use of the $x$-rays; and in neurology, electro-therapeutics, orthopædics, intestinal surgery, and gynæcology. In each class the numbers are limited.

Tutorial classes for practitioners reading for the higher examinations have recently been instituted, and have proved most successful. Particulars as to fees, hours, etc., can be obtained upon application to the Medical Superintendent, 22 Chenies Street, Gower Street, W.C.

### THE LONDON SCHOOL OF CLINICAL MEDICINE (POST-GRADUATE).

### The Seamen's Hospital, Greenwich.

This post-graduate school under the auspices of the Seamen's Hospital Society is carried on at the Dreadnought Hospital, Greenwich, where an increasing number of students has entered during the past medical year; already many hundreds of qualified practitioners and medical officers in the Services and abroad have attended the courses. The clinical requirements of the school are supplied principally from the wards of the hospital at Greenwich, but there are affiliated to the school the Royal Waterloo Hospital for Women and Children, the Bethlem Hospital for Mental Diseases, and the York Road Lying-in Hospital, where students can study in these special departments. Students of the school are also accepted at the Royal Eye Hospital, Southwark, under special terms.

The laboratories are equipped with the latest scientific requirements, the library is well supplied, and there is good residential accommodation in the neighbourhood.

The Dreadnought Hospital contains 250 beds continuously occupied by cases of wide and varying clinical interest. The admissions differ from those at most other hospitals, in that cases of tuberculosis and venereal disorder are admitted.

The out-patient department has been reorganised and equipped with the latest modern requirements. Besides the ordinary medical and surgical consulting-rooms, it provides special departments for diseases of the eye, diseases of the skin, and diseases of the ear, throat, and nose. There is a large and admirably-arranged operating theatre for in-patients, and a smaller one in the out-patient department. There are also two laboratories, a pathological museum, and post-mortem rooms, where pathology and operative surgery are taught. In the pathological department arrangements have been made whereby investigations of all sorts—chemical, microscopical, biological, etc.—are undertaken at moderate fees. Men engaged in practice in the neighbourhood of the hospital have been invited to join the School as associates at a nominal fee, and a considerable number have already availed themselves of the privilege. By this plan it is possible for those within reach to visit the hospital and attend the cliniques when it is convenient for them to do so—a most excellent arrangement for busy men.

### NORTH-EAST LONDON POST-GRADUATE COLLEGE.

This School, which is in connection with the Prince of Wales's General Hospital, Tottenham, N., is recognised by the University of London, the Admiralty, the India Office, and the University of Cambridge in regard to bacteriology for the D.P.H. diploma as a place of post-graduate study. Opportunities are here afforded for attending demonstrations on various branches of medicine, surgery, and gynæcology, diseases of the eye, ear, throat, nose, skin, fevers, diseases of children, psychological medicine, anæsthetics, *x*-rays, bacteriology, and dentistry. Special classes with a limited attendance have been arranged for these and other subjects. The fee for a three-months' course in any single department is one guinea, or three guineas admits for a similar term to the whole practice of the hospital. A perpetual ticket costs five guineas. Additional information, with syllabus of lectures and special classes, from the Dean.

